# Machine learning approach for discrimination of genotypes based on bright-field cellular images

**DOI:** 10.1038/s41540-021-00190-w

**Published:** 2021-07-21

**Authors:** Godai Suzuki, Yutaka Saito, Motoaki Seki, Daniel Evans-Yamamoto, Mikiko Negishi, Kentaro Kakoi, Hiroki Kawai, Christian R. Landry, Nozomu Yachie, Toutai Mitsuyama

**Affiliations:** 1 Artificial Intelligence Research Center, National Institute of Advanced Industrial Science and Technology (AIST), Tokyo, 135-0064 Japan; 2 AIST-Waseda University Computational Bio Big-Data Open Innovation Laboratory (CBBD-OIL), Tokyo, 169-8555 Japan; 3 Graduate School of Frontier Sciences, The University of Tokyo, Chiba, 277-8561 Japan; 4 Research Center for Advanced Science and Technology, The University of Tokyo, Tokyo, 153-8904 Japan; 5 Institute for Advanced Biosciences, Keio University, Tsuruoka, 997-0035 Japan; 6 Systems Biology Program, Graduate School of Media and Governance, Keio University, Fujisawa, 252-0882 Japan; 7 Research and Development Department, LPIXEL Inc., Tokyo, 100-0004 Japan; 8 Institut de Biologie Intégrative et des Systémes, Université Laval, Québec, QC G1V 0A6 Canada; 9 Département de Biochimie, Microbiologie et Bio-informatique, Faculté de sciences et génie, Université Laval, Québec, QC G1V 0A6 Canada; 10 PROTEO, le regroupement québécois de recherche sur la fonction, l’ingénierie et les applications des protéines, Université Laval, Québec, QC G1V 0A6 Canada; 11 Centre de Recherche en Données Massives (CRDM), Université Laval, Québec, QC G1V 0A6 Canada; 12 Département de Biologie, Faculté des sciences et de Génie, Université Laval, Québec, QC G1V 0A6 Canada; 13 School of Biomedical Engineering, The University of British Columbia, Vancouver, V6T1Z3 Canada

**Keywords:** Computational biology and bioinformatics, Microscopy

## Abstract

Morphological profiling is a combination of established optical microscopes and cutting-edge machine vision technologies, which stacks up successful applications in high-throughput phenotyping. One major question is how much information can be extracted from an image to identify genetic differences between cells. While fluorescent microscopy images of specific organelles have been broadly used for single-cell profiling, the potential ability of bright-field (BF) microscopy images of label-free cells remains to be tested. Here, we examine whether single-gene perturbation can be discriminated based on BF images of label-free cells using a machine learning approach. We acquired hundreds of BF images of single-gene mutant cells, quantified single-cell profiles consisting of texture features of cellular regions, and constructed a machine learning model to discriminate mutant cells from wild-type cells. Interestingly, the mutants were successfully discriminated from the wild type (area under the receiver operating characteristic curve = 0.773). The features that contributed to the discrimination were identified, and they included those related to the morphology of structures that appeared within cellular regions. Furthermore, functionally close gene pairs showed similar feature profiles of the mutant cells. Our study reveals that single-gene mutant cells can be discriminated from wild-type cells based on BF images, suggesting the potential as a useful tool for mutant cell profiling.

## Introduction

Recent advancements in machine vision added more values to microscopy by making images machine readable, which is a crucial factor for enabling a high-throughput image-based screening at single-cell resolution^1^. Cell imaging with machine vision automatically extracts hundreds of morphological features from a cell image and enables multivariate analyses to characterize various cell samples such as genetically perturbed cells, differentiations of living cells, and drug-treated cells^2–4^. These phenotyping approaches are known as morphological profiling (or image-based profiling), which shows successful results in variable applications such as identifying phenotypes specific to diseases, novel functions of genes, and modes of drug action^5,6^. Behind these promising results, the details of morphological features are not scrutinized. Moreover, the relationship between a morphological feature and a genotype is not fully understood.

In terms of imaging for morphological profiling, fluorescent microscopy images targeting specific organelles are broadly used^7–9^, whereas bright-field (BF) microscopy images of label-free cells are used in few works. This is because BF images of label-free cells show a lower contrast, and are relatively difficult to be used for characterizing single cells than phase-contrast, differential interference contrast (DIC), or fluorescent imaging. However, BF microscopy still has several advantages. First, BF microscopy can image cells in a natural state in terms of sample preparation because it does not require staining with dyes, which can sometimes be toxic. Second, BF microscopy conveys information related to multiple organelles but not specific organelles. Third, BF microscopy is less subject to artifacts such as shade-off and halo which are found in phase-contrast imaging, and less expensive than DIC imaging which requires special objective lenses and filtering systems. Thus, single-cell profiling based on BF microscopy will enable more efficient and lower-cost phenotyping.

Previous studies demonstrated that BF image-based profiling successfully discriminates different cell lines^10^, infected/noninfected macrophages, and live/dead cells^11^. Also, BF images contained the information of localization or morphology of organelles^12–14^. Another potential use of BF image-based profiling would be to discriminate mutants from wild-type cells and discriminate among mutants in different genes. However, it remains to be tested whether the effects of single-gene perturbation can be evaluated based on BF images.

In this study, we examine whether known morphological features can identify genetically modified cells from the wild type. CRISPR-Cas9 genome editing was performed to obtain genetically modified cells and to acquire BF images of modified/wild-type cells to interrogate. We targeted the ubiquitin-proteasome complex, which is crucial for protein degeneration. The complex consists of multiple pairs of paralogous genes (Table 1) that make the effect of single-gene silencing nonlethal and limited. Possibly, they show zero or minimal morphological effects in a cellular image. With these configurations to have a minimal effect of genetic perturbations and morphological changes, the acquired BF images were processed computationally to extract hundreds of morphological features. By using these numerical features and a machine learning method, we conducted a classification test to distinguish mutant cells from the wild type. Interestingly, the mutants were successfully discriminated from the wild type (area under the receiver operating characteristic curve (AUC) = 0.773). We investigated the morphological features that contributed to the discrimination. Furthermore, it was found that the mutants of functionally close genes share similar feature profiles.

**Table 1:** List of the target genes for creating mutant cells.

Target gene	Functional information
PSMA2	Subunit alpha-2 of the alpha ring of the proteasome core complex. Paralog of PSMA7.
PSMA7	Subunit alpha-4 of the alpha ring of the proteasome core complex. Paralog of PSMA2.
PSMB5	Subunit beta-5 of the beta ring of the proteasome core complex. Paralog of PSMB6.
PSMB6	Subunit beta-1 of the beta ring of the proteasome core complex. Paralog of PSMB5.
PSME1	Alpha subunit of the PA28 complex involved in the immunoproteasome. Paralog of PSME2.
PSME2	Beta subunit of the PA28 complex involved in the immunoproteasome. Paralog of PSME1.
UBQLN1	Ubiqulin that shuttles a protein to the proteasome. Paralog of UBQLN2.
UBQLN2	Ubiqulin that shuttles a protein to the proteasome. Paralog of UBQLN1.

## Results

### BF image dataset of single-gene mutant cells and wild-type cells

**Fig. 1:**
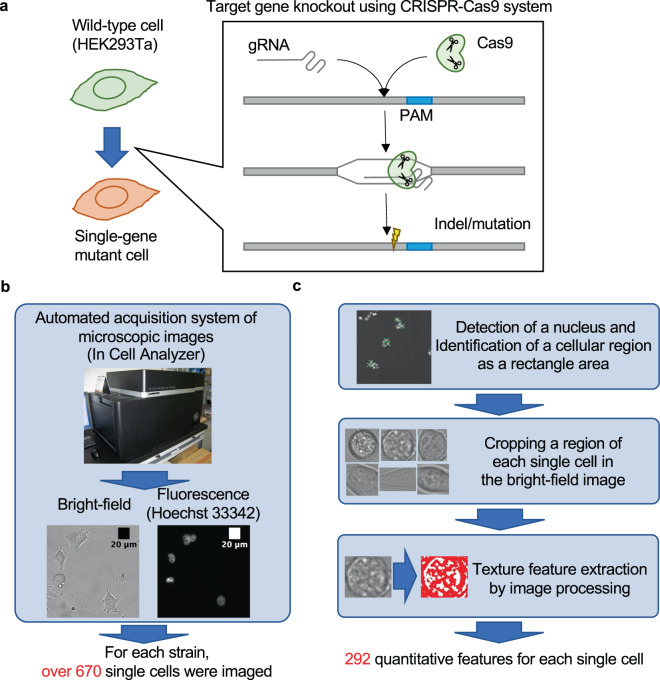
Workflow for the image analysis of single-gene mutant cells. **a** HEK293Ta was used as parental cells (wild type), and single-gene knockout mutant cells were produced by CRISPR-Cas9 genome editing. **b** Cell imaging. Using an automated image acquisition system, the BF image and the fluorescent image (Hoechst33342) of over 670 cells were obtained for each mutant and the wild type. **c** Quantification of texture features for each single cell. Each single-cell region was identified based on the position of a nucleus, and texture features were quantified from the BF images.

To examine whether single-gene mutant cells can be discriminated from wild-type cells in BF images, we constructed a workflow to quantify the features of every single cell as shown in Fig. 1. We prepared single-gene mutants using the CRISPR-Cas9 system (Fig. 1a). We selected eight nonlethal target genes involved in the ubiquitin-proteasome system. These genes have functionally redundant paralogs, which makes them nonessential (Table 1). BF and fluorescent images for every single cell with a stained nucleus were acquired with a high-throughput image acquisition system. Over 670 single cells were imaged for each mutant and the wild type (Fig. 1b). A bounding box surrounding a cell in the BF images was determined using the position of spotted nucleus in the fluorescent images, and features corresponding to textures were quantified by image processing (Fig. 1c). Finally, we quantified 296 texture features for every single cell (see “Methods”; raw data are available as Supplementary Data 1–3).

### Machine learning for discriminating genotypes based on BF cellular images

**Fig. 2:**
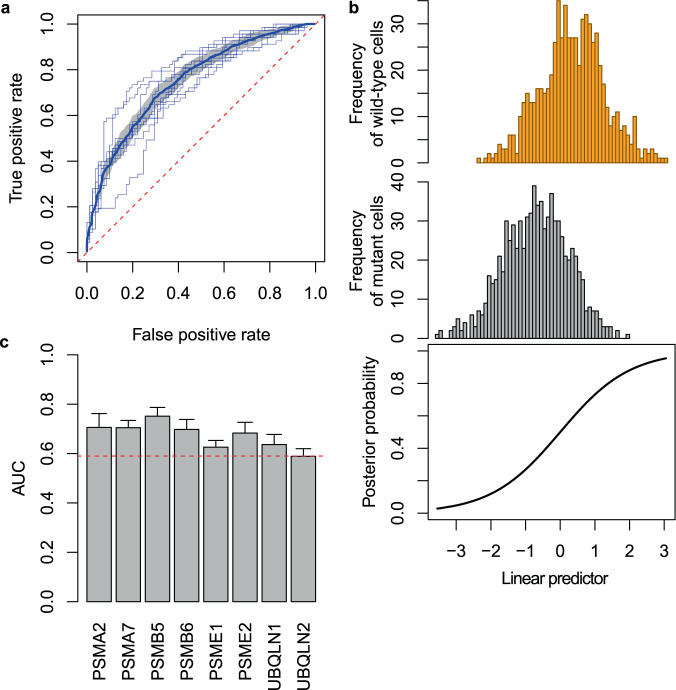
Evaluation of the discriminative models for mutant cells. **a** Receiver operating characteristic (ROC) curves in the discrimination of PSMB5 mutant cells from wild-type cells. Thin blue lines represent ROC curves calculated with tenfold cross-validation. Thick blue line and gray area represent the mean and the quartile of the ROC curves, respectively. **b** Relationship between posterior probabilities and linear predictors of the discriminative model for PSMB5 mutant cells. Upper and middle plots show the distributions of linear predictors of wild-type cells and mutant cells, respectively. Lower plot shows a sigmoid curve between posterior probabilities and linear predictors. **c** AUC of the discriminative model for each mutant cell type. The error bar shows the standard deviation in tenfold cross-validation. Red dashed line represents AUC = 0.59.

We employed logistic regression as a machine learning model to discriminate mutants from wild-type cells using 296 texture features. To identify a subset of the features that contributed to the discrimination, we used the logistic regression with feature selection by L1 regularization (see “Methods”). As an example, the results of the discrimination of PSMB5 mutant cells were shown in Fig. 2a, b. The performance of the discrimination was evaluated by AUC with tenfold cross-validation (the penalty parameter in L1 regularization was optimized by nested cross-validation; see “Methods”). The mean AUC was 0.773 (Fig. 2a; the evaluation measures other than AUC are reported in Supplementary Table 1), which indicated that the mutant cells can be discriminated from wild-type cells compared to the random guessing baseline (i.e., AUC = 0.5). The distributions of linear predictors (see “Methods”) were different between the mutant cells and wild-type cells while overlapping partly (Fig. 2b). The discriminative models for the other mutant cell types were constructed in a similar way, achieving AUC larger than 0.59 (Fig. 2c). These results demonstrate that BF images contain information for discriminating mutant cells from wild-type cells.

In addition to logistic regression with L1 regularization, we tested other machine learning models including support vector machine (SVM), random forest, and k-nearest neighbor with and without dimensionality reduction by principal component analysis (PCA) (see “Methods”). The all models showed comparable AUC while logistic regression with L1 regularization achieved the highest value (Supplementary Table 2). We also evaluated the effects of different image preprocessing methods (blur, edge, and sharp) on the performance of the discriminative model (see “Methods”), and found that AUC was slightly decreased by these preprocessing methods (Supplementary Table 3).

### Features of BF cellular images that contributed to the discrimination

**Fig. 3:**
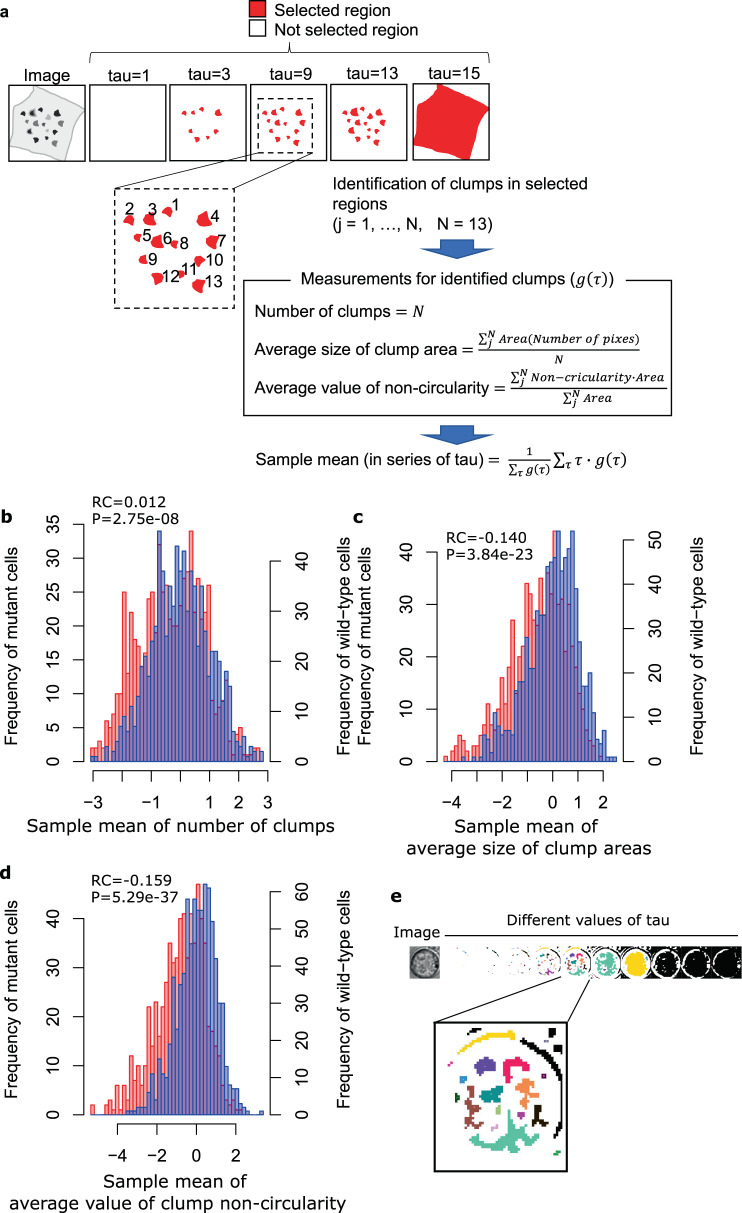
Examples of the large-weight features in the discriminative model for PSMB5 mutant cells. **a** Schematics of the feature extraction procedure from BF images. Tau indicates a threshold of pixel value to identify clumps within a cellular region as described previously^15^. **b** Distribution of PSMB5 mutant cells and wild-type cells on the feature “sample mean of number of clumps”. Blue and red histograms represent mutant and wild-type cells, respectively. *P* value of one-sided *U* test between mutant and wild-type cells, and the regression coefficient (RC) in the logistic regression model are shown. **c** Distribution of PSMB5 mutant cells and wild-type cells on the feature “sample mean of average size of clump areas” shown in the same way as in **b**. **d** Distribution of PSMB5 mutant cells and wild-type cells on the feature “sample mean of average value of noncircularity” shown in the same way as in **b**. **e** Example of clump detection using an image of a wild-type cell. The leftmost panel is an input cellular image, and the right panels are those processed with different thresholds. In the magnified panel, each clump is shown in a different color.

What kind of biological information was included in the features which contributed to the discriminative models for mutant cells? To address this question, we focused on the features that had large weights in the discriminative models (i.e., features selected by L1 regularization). We found that large-weight features included those calculated from “clumps” observed in BF images, which possibly represent organelles and other intracellular structures (e.g., nucleus, nucleolus, and parts of nuclear envelop) as suggested in a previous study^13^. For example, the three large-weight features^15^ in the discriminative model for PSMB5 mutant cells quantify the number or morphology of darker clumps (Fig. 3a, e). First, regions with darker pixels were selected by thresholds. Next, connected regions were identified as clumps, and measurements (e.g., number, size, and shape) were calculated from the identified clumps. Finally, the sample mean was calculated from the measurements of different thresholds. The PSMB5 mutant cells showed a larger number of darker clumps compared with wild-type cells (Fig. 3b). In addition, clumps with larger size (Fig. 3c) and more irregular (noncircular) shape (Fig. 3d) were also observed in the PSMB5 mutant cells than in wild-type cells. These results show that the BF images captured the morphological changes in the mutant cells. Supplementary Fig. 1 shows other examples of features contributed to the discrimination of PSMB5 mutant cells. These features included the statistics computed from pixel intensity distributions (Supplementary Fig. 1a) and various shape-related measurements such as thinness, size, and perimeter after binarization (Supplementary Fig. 1b–d).

**Fig. 4:**
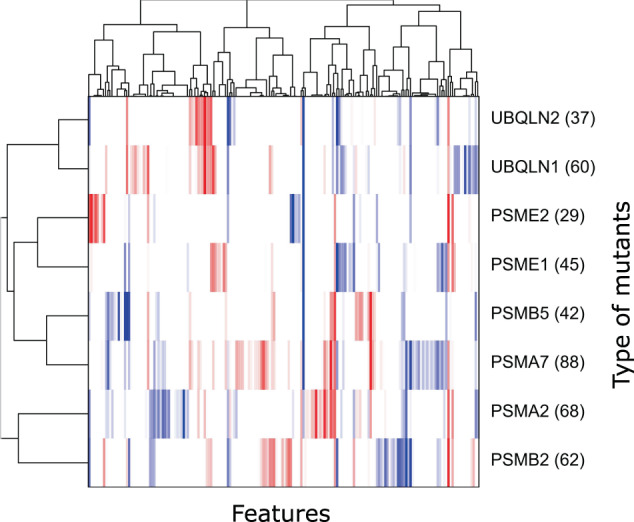
Comparison of the mutants based on morphological profiles. Clustering of mutants (vertical) and features (horizontal) using the regression coefficients in the discriminative models. Red and blue colors in the heatmap represent positive and negative regression coefficients, respectively. Features not selected by L1 regularization were colored in white. Numbers described on the right side of gene names show the number of selected features.

On average, 53 features were selected for the discriminative model for each mutant by the L1 regularization in the logistic regression (Fig. 4). This analysis indicated that there are multiple traits by which mutant cells can be discriminated from wild-type cells (number of features in Fig. 4). On the other hand, the profiles of contributed features for the discriminative models were distinct among the mutants (heatmap in Fig. 4).

If morphological changes in mutant cells were caused by the functional deficiency of a target gene, mutants of functionally similar genes are expected to show similar morphological profiles. To examine this hypothesis, we performed hierarchical clustering based on the similarity of the feature profiles (i.e., regression coefficients in the discriminative model). As expected, paralog pairs (PSME1 and PSME2; UBQLN1 and UBQLN2), which have similar functions, were classified in the same clusters (Fig. 4). PSME1 and PSME2, respectively, encode the alpha and beta subunit of PA28, a component of the immunoproteasome^16^. Therefore, the similarity of feature profiles between these mutants may represent the common dysfunction of the immunoproteasome caused by the mutations. UBQLN1 and UBQLN2 both encode a shuttling protein that brings ubiquitinated proteins to the proteasome while their functional differences remain elusive^17^. The similarity of their feature profiles may suggest this functional similarity. Interestingly, a nonparalog pair (PSMB5 and PSMA7) was classified in the same cluster (Fig. 4). We speculate that this is associated with the proximity of encoded proteins in the proteasome core complex. Specifically, PSMB5 and PSMA7, respectively, encode the subunit beta-5 and alpha-4 that directly contact in the proteasome core complex, while it is not the case for proteins encoded by PSMA2 and PSMB6^18^. In summary, these results suggest that the similarity of morphological changes in the mutant cells represent not only the functional similarity of mutated genes but also the physical interaction of their encoded proteins.

We also explored the correlation between types of mutations and features rather than individual mutations and features (Supplementary Table 4). The eight mutant cells were grouped into four types based on their paralogous relationship (PSMA2/7, PSMB5/6, PSME1/2, and UBQLN1/2). For each paralog pair, we extracted specific features in the discriminative models (e.g., features with positive regression coefficients in the discriminative models of PSMA2 and PSMA7 mutant cells, but zero or negative regression coefficients in the discriminative models of the other mutant cells). The number of features detected in each type of mutants was relatively small, suggesting that group-to-group relationships between mutants and features are not so strong.

## Discussion

The objective of the present study was to investigate the potential ability of BF microscopy cell images for classifying single-gene mutants. We demonstrated that the BF images of the single-gene mutants could be discriminated from those of wild types. In addition, morphological features of structures detected within cellular regions contributed to the discrimination, and gene pairs sharing common functions showed similar feature profiles in mutant cells.

We showed that single-gene mutant cells could be discriminated from wild-type cells based on BF images. Only a few studies performed the classification of cells based on BF images before. They classified multiple cell lines^10^ or discriminated between infected and noninfected macrophages, and between living and dead macrophages^11^. The problems addressed in these previous studies were the classification of cells with largely different characteristics: cell lines harboring different alleles in multiple loci as in ref. ^10^, and different forms of macrophages harboring changes in a cellular process involving multiple genes (infection and cell death) as in ref. ^11^. In contrast, our study showed that BF images can also be used for discriminating single-gene perturbation.

We observed that the discriminative models of mutant cells may be related with organelles. As shown in Fig. 3, features calculated from darker clumps within cellular regions contributed to the discrimination. In a previous study^13^, darker clumps of cellular regions were used as a morphological feature for analyzing the dynamics of organelles. Other studies also showed that BF images contain information on the morphologies of organelles: cellular nuclei could be detected in BF images^11^, and the localization and morphology of multiple organelles could be predicted simultaneously from BF images by machine learning trained with fluorescent images as label data^19^. Therefore, we considered that the discriminative models we constructed may have learned the pattern of morphological changes of organelles in mutant cells.

The similarity between feature profiles of mutants was associated with the functional similarity of target genes (Fig. 4). This suggests that feature profiles used in the present study may reflect the effects of the dysfunction of target genes. A similar result was observed in a previous study^19^ where the morphological analysis of nuclear core complex subunits was performed using fluorescent microscopy. Our study shows that the information of the dysfunction of target genes could also be extracted from BF images.

We demonstrated that mutant cells could be analyzed based on BF images using a simple machine learning model. In previous studies, the morphological analysis of organelles based on BF microscopy used a special high-resolution microscope equipment^12^ or 3D-stacked images^19^. In another previous study^10^, the discrimination between different cell types based on BF microscopy was performed using convolutional neural network (CNN) trained with over 8000 images for each cell type. In the present study, we performed the discrimination of mutant cells using a simpler machine learning model (logistic regression) trained with smaller training data (>670 nonstacked 2D images).

To investigate the influence of training data size, we performed a computational experiment where the discriminative model was trained using a subset of the whole training data (Supplementary Fig. 2a). We found that the increase of AUC with the increase of training data size was almost saturated except for the UBQLN1 and UBQLN2 mutant cells, suggesting that the further increase of training data size will not improve AUC largely. For UBQLN1 and UBQLN2 mutant cells, AUC may be improved by increasing training data size. We note that the numbers of the whole training data for UBQLN1 and UBQLN2 were larger than the other mutant cells (Supplementary Fig. 2b).

We evaluated the reproducibility by utilizing the three datasets that differed in the numbers of cells initially seeded in a well: C10000, C2000, and C400 (see “Methods”). Specifically, the discriminative model was trained using the C10000 dataset, and its performance was evaluated using C2000 and C400 datasets as independent fold out. The results are summarized in Supplementary Table 5. We found that AUC values on C2000 and C400 were slightly lower than that with the cross-validation on C10000. Nonetheless, they were higher than the random guessing baseline (AUC = 0.5) in the all mutant cells. These results suggest that our discriminative models had a certain level of the applicability on independent datasets, and thus the reproducibility of our study.

We acknowledge that the present study has limitations as follows. First, there were possibilities of off-target mutations caused by CRISPR-Cas9 genome editing. To deal with this problem, we pooled the datasets of two different mutant clones for each target gene. However, to evaluate the effect of possible off-target mutations, we need to perform whole genome sequencing of mutant clones. Second, we only analyzed mutants of genes involved in the ubiquitin-proteasome system. We need to analyze more mutants of genes related to different cellular pathways or functions.

The present study demonstrated that mutant cells can be discriminated by BF images in combination with machine learning. This finding has a broad impact on biomedical research since it suggests that genetically altered cells can be detected without labeling. In this regard, other types of nonlabel imaging such as DIC and phase-contrast microscopy will be useful to provide additional training data for improving the accuracy of machine learning prediction.

## Methods

### Cells and culture

We established Cas9-expressing 293Ta cells by transducing lentiCas9-blast to human embryonic kidney (HEK) 293Ta cells (GeneCopoeia) and maintained in Dulbecco’s modified Eagle’s medium (Sigma) supplemented with 10% fetal bovine serum (Thermo Fisher Scientific), 5 µg/mL of blasticidin S (Kaken Pharmaceutical), and penicillin–streptomycin (Wako) to use as a parental strain. Each of eight genes involved in the ubiquitin-proteasome system was selected as a target gene and perturbed by the following procedures. Plasmids encoding single-guide RNA targeting each of the eight genes (Supplementary Table 6) were constructed by inserting annealed oligonucleotides into a derivative of lentiGuide-Puro (Addgene 52963), named lentiTRACE-puro, which has a guide RNA expressing module in 3′LTR, as previously reported^20^. To produce lentiviral particles, 500 ng of sgRNA-encoding lentiTRACE-puro, 375 ng of psPAX2 (Addgene 12260), and 125 ng of pMD2.G (Addgene 12259) were cotransfected to 2 × 10^5^ cells of HEK293Ta with 3 µL of 1 mg/mL polyethyleneimine MAX (Polysciences). On the next day of transfection, the culture media were replaced with fresh media and the supernatant containing lentiviral particles was harvested after 2 days of incubation. To transduce an sgRNA, 1 × 10^5^ cells/well of Cas9-expressing 293Ta cells, which were seeded in a 12-well plate on the day before infection, were incubated in 500 µL of the lentiviral supernatant with 8 µg/mL of polybrene (Sigma). The infected cells were selected by 2 µg/mL of puromycin (Thermo Fisher Scientific) from the next day of transfection. After 20 days, cells were isolated by FACS Jazz cell sorter (BD Biosciences) as a single cell/well in a 96-well plate. Following the passage of the grown cells to three of 96-well plates, genomic DNA was extracted by boiling in 50 µL of 50 mM sodium hydroxide at 95 °C for 15 min and neutralizing by adding 5 µL of 1 M Tris-HCl (pH 8.0). The genotyping sequencing library was prepared by sequential PCRs with row-column-plate-PCR (RCP-PCR) primer sets^21^. Briefly, the first PCR was performed with gene-specific genotyping primers for every plate (Supplementary Table 6), followed by purification by Agencourt AMPure XP beads (Beckman Coulter) following the manufacturer’s instruction. Purified PCR products were combined in a single 96-well plate with keeping their well-position and used for the second PCR reaction with row- and column-tagged forward and reverse primers, respectively (Supplementary Table 6). The second PCR products were collected in a single tube and purified by gel extraction with FastGene Gel/PCR Extraction Kit (Nippon Genetics) to be used for the third PCR to attach P5 and P7 adapters for Illumina sequencing on 5′ and 3′ ends of the fragments, respectively (Supplementary Table 6). After gel extraction of the PCR products, the genotyping libraries were quantified by KAPA Library Quantification Kit (KAPA Biosystems) and sequenced with paired-end reads by Illumina MiSeq using MiSeq Reagent Kit v3 (Illumina). The obtained sequence data were used to identify the genotype of isolated clones. First, RCP-PCR indices and target regions were identified and extracted from each sequencing read for demultiplexing. Simultaneously, the target region of the read was aligned to the wild-type sequence using BLASTn version 2.4.0^22^ with default parameters. Second, the alignment results for demultiplexed reads were aggregated to compute the allele frequency within each of the clone-derived sample. Any allele appearing less than 10% within each demultiplexed sample was eliminated for further analysis to cancel out sequencing error. Finally, the remaining count data of each allele were used to identify the genotype of the isolated clones. Clones having frame-shift mutation on both alleles were used for further analysis (Supplementary Fig. 3). The scripts used for analyzing genotype information from RCP-PCR data are available from the authors’ GitHub website (https://github.com/yachielab/RCP-PCR_CRISPR_KO).

### Image acquisition

HEK293Ta single-gene knockout clones were seeded 400, 2000, and 10000 cells/well in Cellstar 96-well µ-clear plates (Greiner). One, two, and three days after passage, cells were nuclear-stained by 5 µg/mL of Hoechst33342 (Thermo Fisher Scientific) in a CO_2_ incubator for 15 min. Twenty-five fields of cell images per well for BF and nuclear-staining images were obtained by IN Cell Analyzer 6000 (GE Healthcare) with 20× lens and Bright Field-DsRed and UV-DAPI filter sets, respectively. We generated three datasets that differed in the numbers of cells initially seeded in a well: 10,000 cells (C10000), 2000 cells (C2000), and 400 cells (C400). Unless otherwise described, the results reported in this paper were those on C10000 dataset. C2000 and C400 datasets were used for evaluating the applicability of the discriminative model on independent fold-out datasets (Supplementary Table 5).

### Single-cell feature quantification

For single-cell profiling, we quantified 296 texture features from BF images. First, we detected nuclear regions in fluorescent images of cells treated with Hoechst33342 by a machine learning method. In brief, the nuclei detector was developed by using Faster R-CNN with ResNet-101 backbone^23^. We trained the nuclei detector with a part of the Hoechst33342 images in which each of the nuclei is annotated with a bounding box. Then, the nuclei detector was applied to all of the Hoechst33342 images. We cropped a cellular region of BF images based on the position of the rectangle region enclosing the detected nucleus in the Hoechst33342 images. We quantified texture features in the segmented cellular area of BF images using the LPX296 feature extractor (formerly the KBI feature extractor^24^). The BF images used for feature quantification are available as Supplementary Data 1 and 2. The feature values for each BF image are available as Supplementary Data 3.

### Data preprocessing

Each of cellular texture features in BF images was standardized by the mean and standard deviation calculated from the cell population of the wild-type strain. In each mutant cell population and in each of the texture features, we identified outliers and removed them with the cutoff of 3 standard deviations from the mean. After the normalization and removal of outliers, we pooled two datasets of different clones screened independently for each target gene.

### Discriminative model construction

To discriminate between mutant cells and wild-type cells, we built a logistic regression model using R package glmnet^25^. We employed the least absolute shrinkage and selection operator method based on L1 regularization for feature selection. Briefly, the logistic regression model is represented as follows:

1 $${\boldsymbol{y}} = \sigma \left( {\beta ^T{\boldsymbol{x}} + \alpha } \right) + \varepsilon$$

where *y* is the binary variable representing a cell is the mutant or the wild type, *σ* is a sigmoidal function, ***x*** is a 296-dimensional feature vector computed from the cellular image, and *ε* is an error term. Given a set of training data $S = \left\{ {\left( {y,{\boldsymbol{x}}} \right)} \right\}$, the model fits regression coefficients *β* (also referred to as feature weights) and *α* by minimizing the error with the L1 regularization term:

2 $$\mathop {\sum }\limits_{\boldsymbol{S}} \left( {{\boldsymbol{y}} - \sigma \left( {\beta ^T{\boldsymbol{x}} + \alpha } \right)} \right)^2 \,+\, {\lambda}\left\| \beta \right\|_1$$

In Fig. 2, the linear predictor and the posterior probability represent $\beta ^T{\boldsymbol{x}} + \alpha$ and $\sigma \left( {\beta ^T{\boldsymbol{x}} + \alpha } \right)$, respectively, computed from the fitted model. To measure a discrimination accuracy for each model, we calculated AUC using tenfold cross-validation. To optimize the penalty parameter λ in L1 regularization, we performed nested tenfold cross-validation where the validation dataset in the inner loop was used for selecting λ, and the test dataset in the outer loop was used for measuring the model accuracy.

### Comparison of various machine learning models and preprocessing methods

To compare various machine learning models with logistic regression, we tested SVM, random forest, and k-nearest neighbor with and without PCA on the PSMB5 dataset. These models were implemented with Python package scikit-learn. The hyperparameters of each model were optimized by nested tenfold cross-validation as in the case of logistic regression. To evaluate the effect of various preprocessing methods, we used “BLUR”, “EDGE_ENHANCE,” and “SHARPEN” functions in Python PIL package.

### Hierarchical clustering of mutants based on morphological similarity

The similarity between mutants was evaluated by Pearson’s correlation coefficient of their morphological profiles. As the morphological profile of a mutant, we employed regression coefficients (beta value) of features in the logistic regression model. Complete linkage clustering was conducted using hclust function in R.

### Reporting summary

Further information on research design is available in the Nature Research Reporting Summary linked to this article.

## Supplementary information

Reporting Summary

Supplementary Information

Supplementary Data 1

Supplementary Data 2

Supplementary Data 3

Supplementary Data 4

Supplementary Data 5

Supplementary Data 6

Supplementary Data 7

Supplementary Data 8

Supplementary Data 9

## Data Availability

All data generated in this study are provided in this paper and its Supplementary Information files.

## Code availability

The codes used for analyzing genotype information from RCP-PCR data are available from the authors’ GitHub website (https://github.com/yachielab/RCP-PCR_CRISPR_KO). The codes used for the other computational analyses are available upon request to the authors.
