# Supplementary material for: Machine learning approach for discrimination of genotypes based on bright-field cellular images
Source: NPJ Syst Biol Appl. 2021 Jul 21;7:31. doi: 10.1038/s41540-021-00190-w (PMC8295336; doi:10.1038/s41540-021-00190-w)

Supplementary Information:

Machine learning approach for discrimination of genotypes based on bright-field cellular images

Godai Suzuki[1], Yutaka Saito[1,2,3], Motoaki Seki[4], Daniel Evans-Yamamoto[4,5,6], Mikiko Negishi[4], Kentaro Kakoi[4], Hiroki Kawai[7], Christian R Landry[8,9,10,11,12], Nozomu Yachie[4,5,6,13,14*], Toutai Mitsuyama[1*]

1. Artificial Intelligence Research Center, National Institute of Advanced Industrial Science and Technology (AIST), Tokyo 135-0064, Japan.
2. AIST-Waseda University Computational Bio Big-Data Open Innovation Laboratory (CBBD-OIL), Tokyo 169-8555, Japan.
3. Graduate School of Frontier Sciences, The University of Tokyo, Chiba 277-8561, Japan.
4. Synthetic Biology Division, Research Center for Advanced Science and Technology, The University of Tokyo, Tokyo 153-8904, Japan.
5. Institute for Advanced Biosciences, Keio University, Tsuruoka 997-0035, Japan.
6. Systems Biology Program, Graduate School of Media and Governance, Keio University, Fujisawa 252-0882, Japan.
7. Research and Development Department, LPIXEL Inc., Tokyo 100-0004, Japan.

8. Institut de Biologie Intégtarive et des Systémes, Université Laval, Québec, QC G1V 0A6, Canada.
9. Département de Biochimie, Microbiologie et Bio-informatique, Faculté de sciences et génie, Université Laval, Québec, QC G1V 0A6, Canada.
10. PROTEO, le regroupement québécois de recherche sur la fonction, l'ingénierie et les applications des protéines, Université Laval, Québec, QC G1V 0A6, Canada.
11. Centre de Recherche en Données Massives (CRDM), Université Laval, Québec, QC G1V 0A6, Canada.
12. Département de Biologie, Faculté de sciences et Génie, Université Laval, Québec, QC G1V 0A6, Canada.
13. Department of Biological Sciences, School of Science, The University of Tokyo, Tokyo 113-0033, Japan.
14. PRESTO, Japan Science and Technology Agency (JST), Tokyo 153-8904, Japan.

* Correspondence to Nozomu Yachie (yachie@synbiol.rcast.u-tokyo.ac.jp); Toutai Mitsuyama (mituyama-toutai@aist.go.jp).

Legends for Supplementary Tables and Supplementary Figures

Supplementary Table 1. The performance of the discriminative models evaluated by various measures. The table is available as a separate Excel file.

Supplementary Table 2. AUC of various machine learning models evaluated for PSMB5 mutant cells. The table is available as a separate Excel file.

Supplementary Table 3. AUC of the discriminative model with various pre-processing methods applied to images, evaluated for PSMB5 mutant cells. The table is available as a separate Excel file.

Supplementary Table 4. Correlation between types of mutations and features. For each paralog pair, specific features in the discriminative models are shown. For example, "PSMA2/7" with "+" means features with positive regression coefficients in the discriminative models of PSMA2 and PSMA7 mutant cells, but zero or negative regression coefficients in the discriminative models of the other mutant cells. The table is available as a separate Excel file.

Supplementary Table 5. AUC of the discriminative model evaluated on independent fold-out datasets C2000 and C400. AUC with the cross-validation on C10000 is shown for comparison. The table is available as a separate Excel file.

Supplementary Table 6. Sequences of the oligos for sgRNA cloning and the primers for library preparation. The table is available as a separate Excel file.

Supplementary Fig. 1. Examples of features contributed to the discrimination of PSMB5 mutant cells. (a-d) Distribution of PSMB5 mutant cells and wild-type cells on each feature. Blue and red histograms represent mutant and wild-type cells, respectively. P-value of one-sided *U*-test between mutant and wild-type cells, and the regression coefficient (RC) in the logistic regression model are shown. A brief description of the algorithm for calculating the feature is described in the right panel. (a) Kurtosis of intensity distribution. (b) Sum of thinness of areas after Sobel and mean filters. (c) Average size of areas after Sobel and 50-percentile filters. (d) Average perimeter of areas after 50-percentile filter. The figure is included in this file.

Supplementary Fig. 2. The effect of training data size on the performance of the discriminative model. (a) AUC with different fractions of training data size. (b) The number of the whole training data. The figure is included in this file.

Supplementary Fig. 3. Overview of the RCP-PCR analysis workflow. The figure is included in this file.

Supplementary Data 1. Bright-field cellular images. Each file contains the image of a cell. The correspondence between the file name, the genotype, and the features of the cell is described in Supplementary Data 3. The data are available as a separate TAR-BZIP file.

Supplementary Data 2. Thumbnail view of bright-field cellular images. For browsing, the images in Supplementary Data 1 are shown as 28×28 thumbnails. The data are available as a separate TAR-BZIP file.

Supplementary Data 3. Feature values used for machine learning. For each cell, the image file in Supplementary Data 1, the genotype, and the features used for machine learning are shown. For the columns whose names start with "X", "X9.x" and "X10.y" represent the coordinates of the cell in the

BF image, and the remaining 296 columns represent features. The data are available as a separate TAR-BZIP file.

Supplementary Fig. 1

a

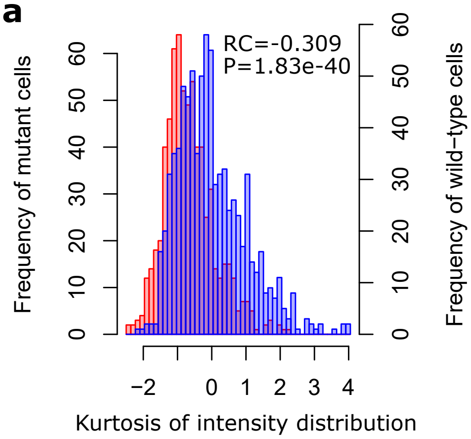

1. compute the intensity distribution of all pixels
2. compute the kurtosis (fourth moment) of the distribution

b

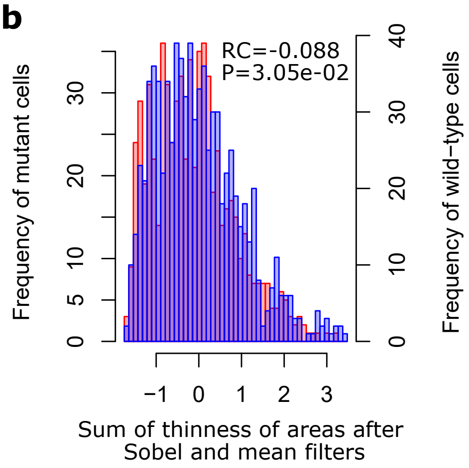

1. perform Sobel filter for edge detection
2. perform binarization with mean filter
3. for each area, compute the thinness (the major axis length divided by the small axis length)
4. compute the sum of thinness of all areas

c

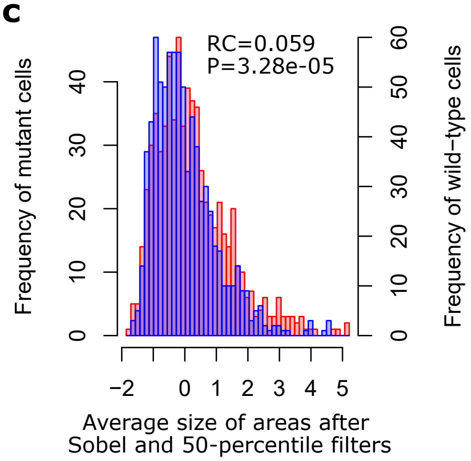

1. perform Sobel filter for edge detection
2. perform binarization with the 50 percentile of intensity
3. for each area, compute the size
4. compute the average size of of all areas

d

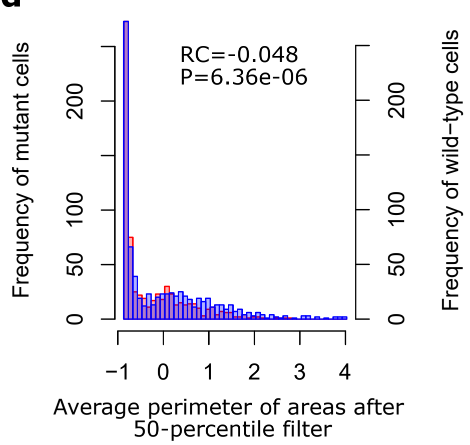

1. perform binarization with the 50 percentile of intensity
2. for each area, compute the perimeter
3. compute the average perimeter of all areas

Supplementary Fig. 2

a

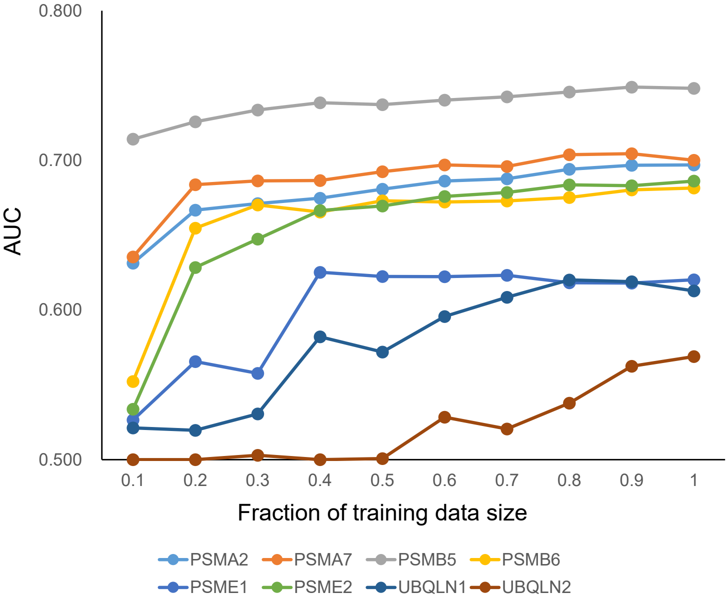

b

Target gene	Training data size	
	Mutant	Wildtype
PSMA2	708	809
PSMA7	904	809
PSMB5	679	809
PSMB6	856	809
PSME1	1138	809
PSME2	1513	809
UBQLN1	1795	809
UBQLN2	1614	809

Supplementary Fig. 3

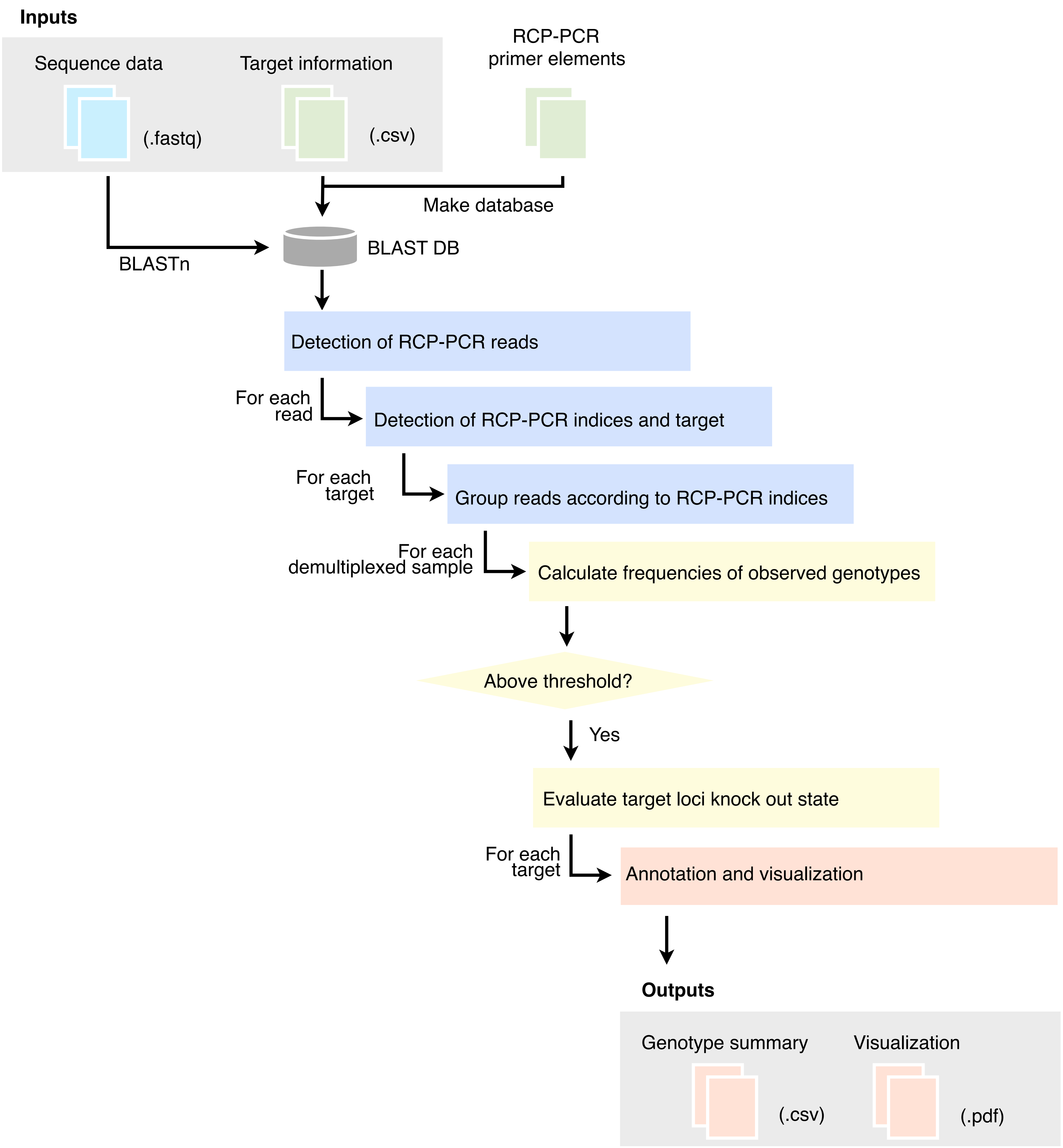

Supplement: Supplementary file 2 — Supplementary Information [file 41540_2021_190_MOESM2_ESM.pdf]
