# Supplementary figures and images for: Machine learning approach for discrimination of genotypes based on bright-field cellular images
Source: NPJ Syst Biol Appl. 2021 Jul 21;7:31. doi: 10.1038/s41540-021-00190-w (PMC8295336; doi:10.1038/s41540-021-00190-w)

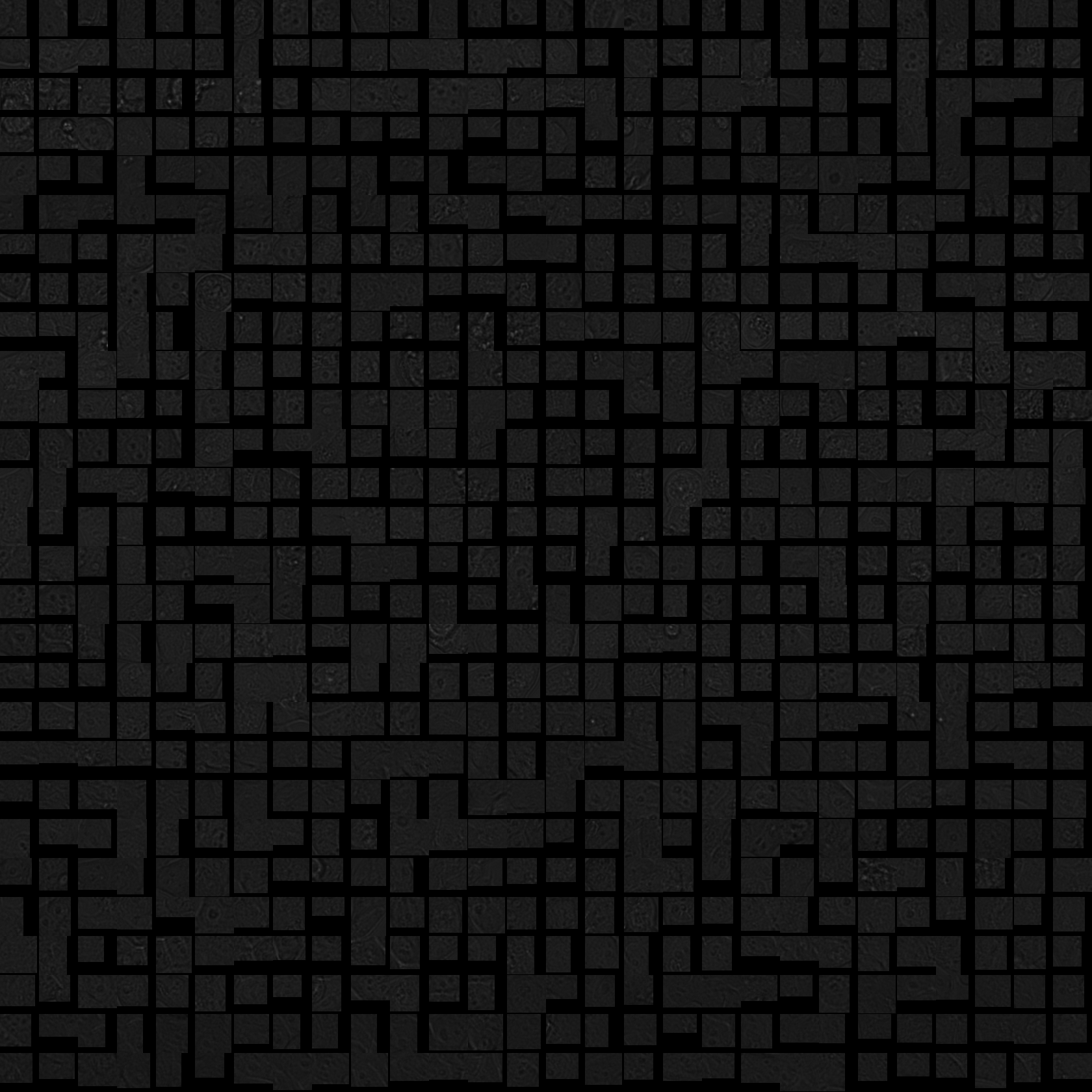

Supplement: Supplementary file 10 — Supplementary Data 8 [file 41540_2021_190_MOESM10_ESM.bz2 › D2_Additional_file_4_SupDataS2/genlarge-01.tif]

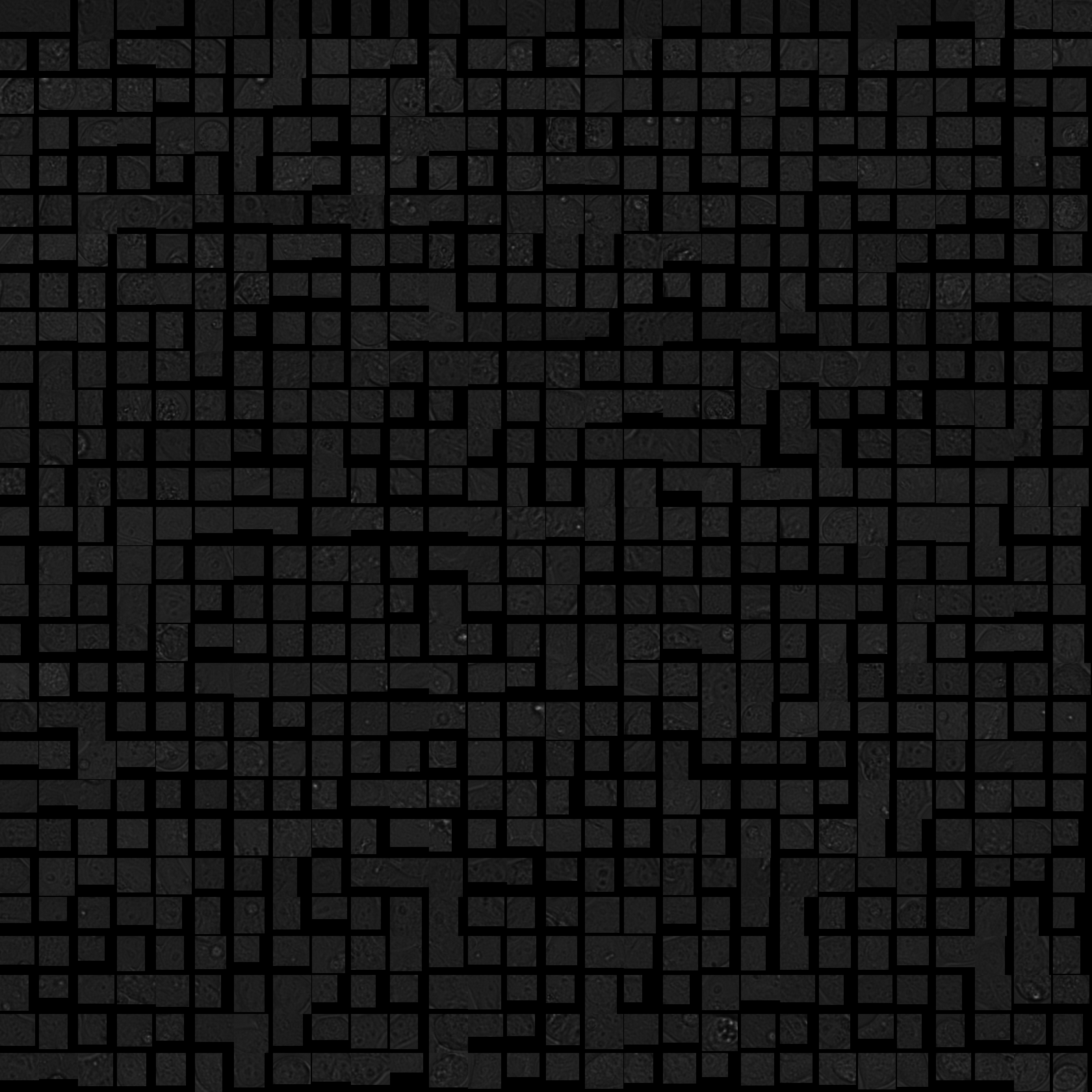

Supplement: Supplementary file 10 — Supplementary Data 8 [file 41540_2021_190_MOESM10_ESM.bz2 › D2_Additional_file_4_SupDataS2/genlarge-02.tif]

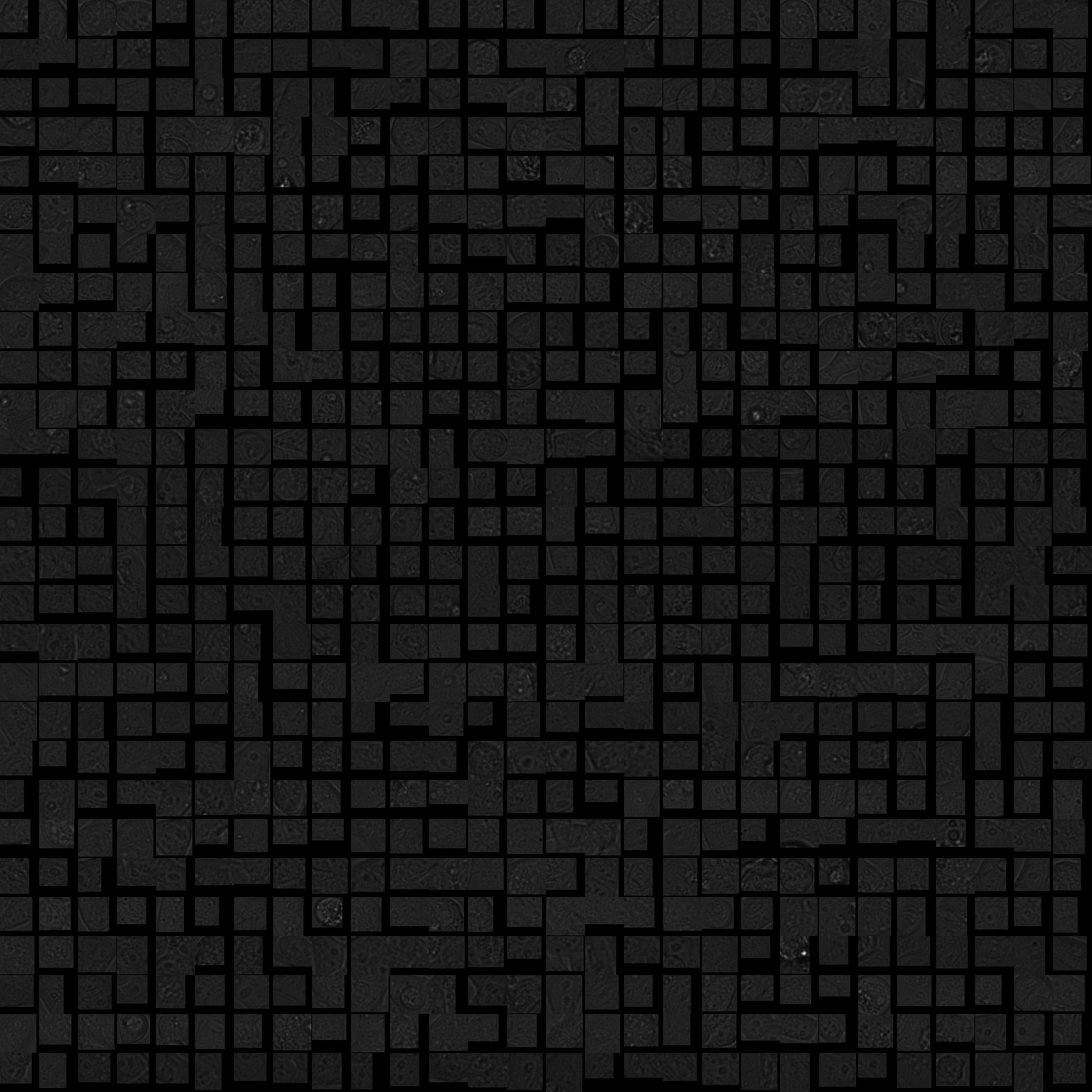

Supplement: Supplementary file 10 — Supplementary Data 8 [file 41540_2021_190_MOESM10_ESM.bz2 › D2_Additional_file_4_SupDataS2/genlarge-03.tif]

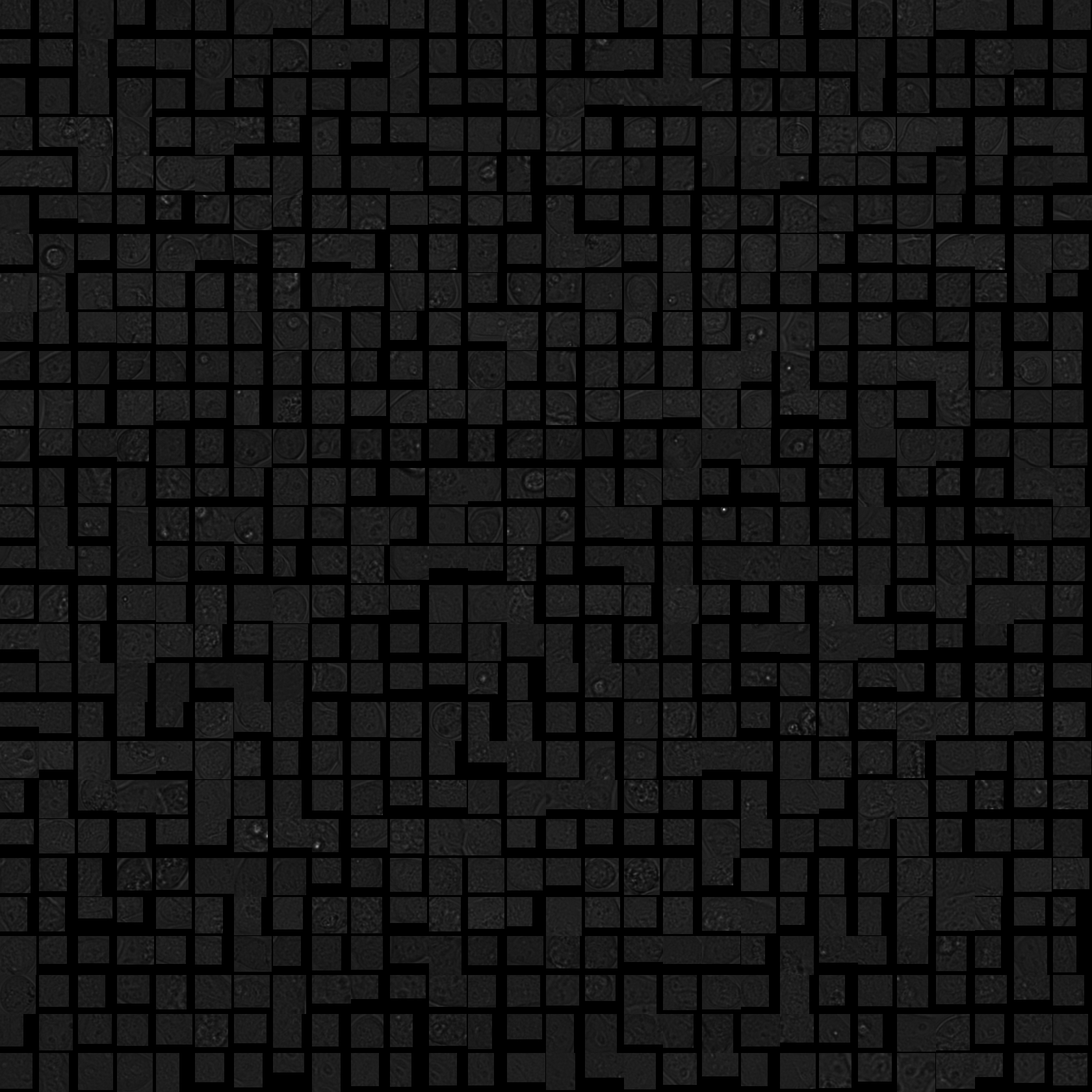

Supplement: Supplementary file 10 — Supplementary Data 8 [file 41540_2021_190_MOESM10_ESM.bz2 › D2_Additional_file_4_SupDataS2/genlarge-04.tif]

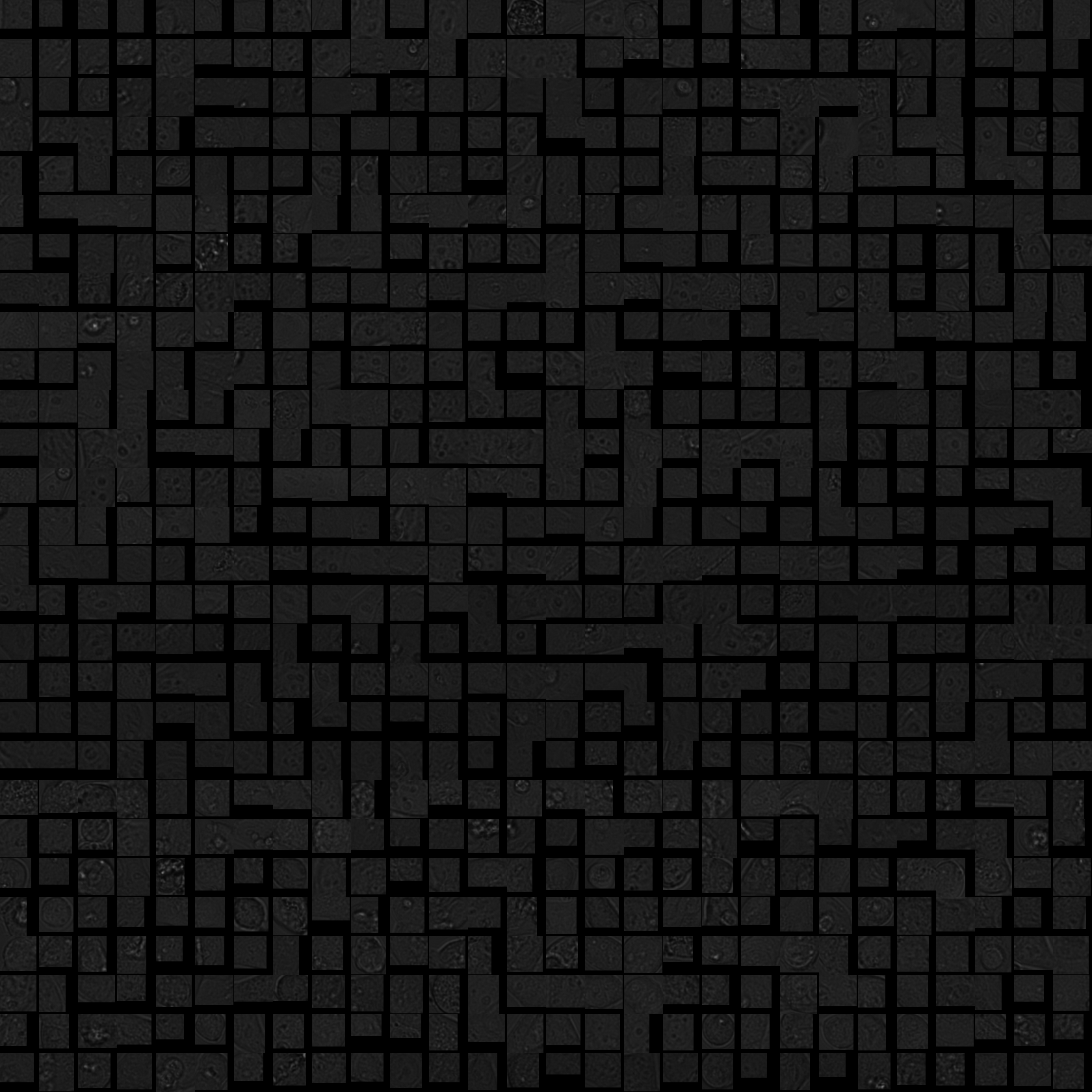

Supplement: Supplementary file 10 — Supplementary Data 8 [file 41540_2021_190_MOESM10_ESM.bz2 › D2_Additional_file_4_SupDataS2/genlarge-05.tif]

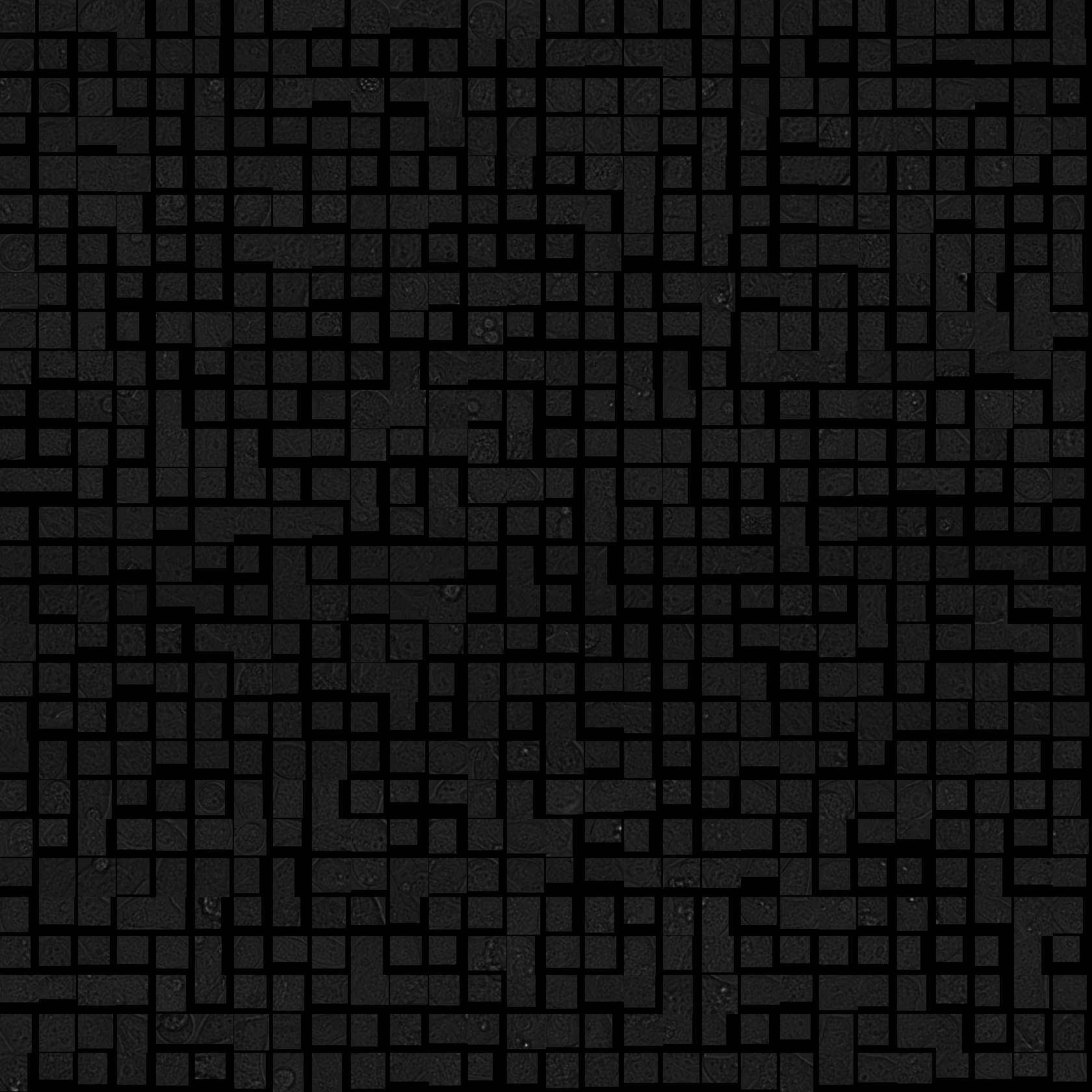

Supplement: Supplementary file 10 — Supplementary Data 8 [file 41540_2021_190_MOESM10_ESM.bz2 › D2_Additional_file_4_SupDataS2/genlarge-06.tif]

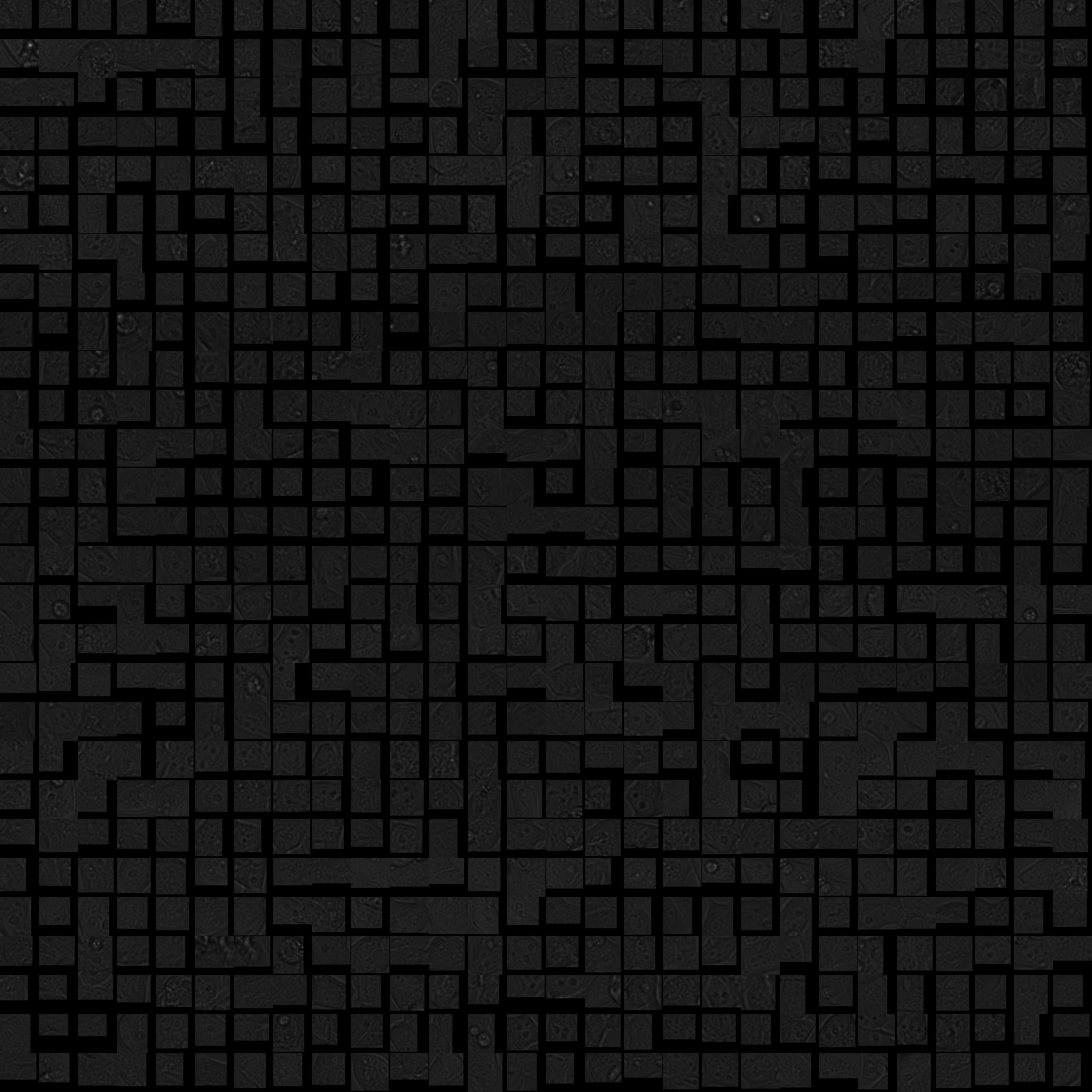

Supplement: Supplementary file 10 — Supplementary Data 8 [file 41540_2021_190_MOESM10_ESM.bz2 › D2_Additional_file_4_SupDataS2/genlarge-07.tif]

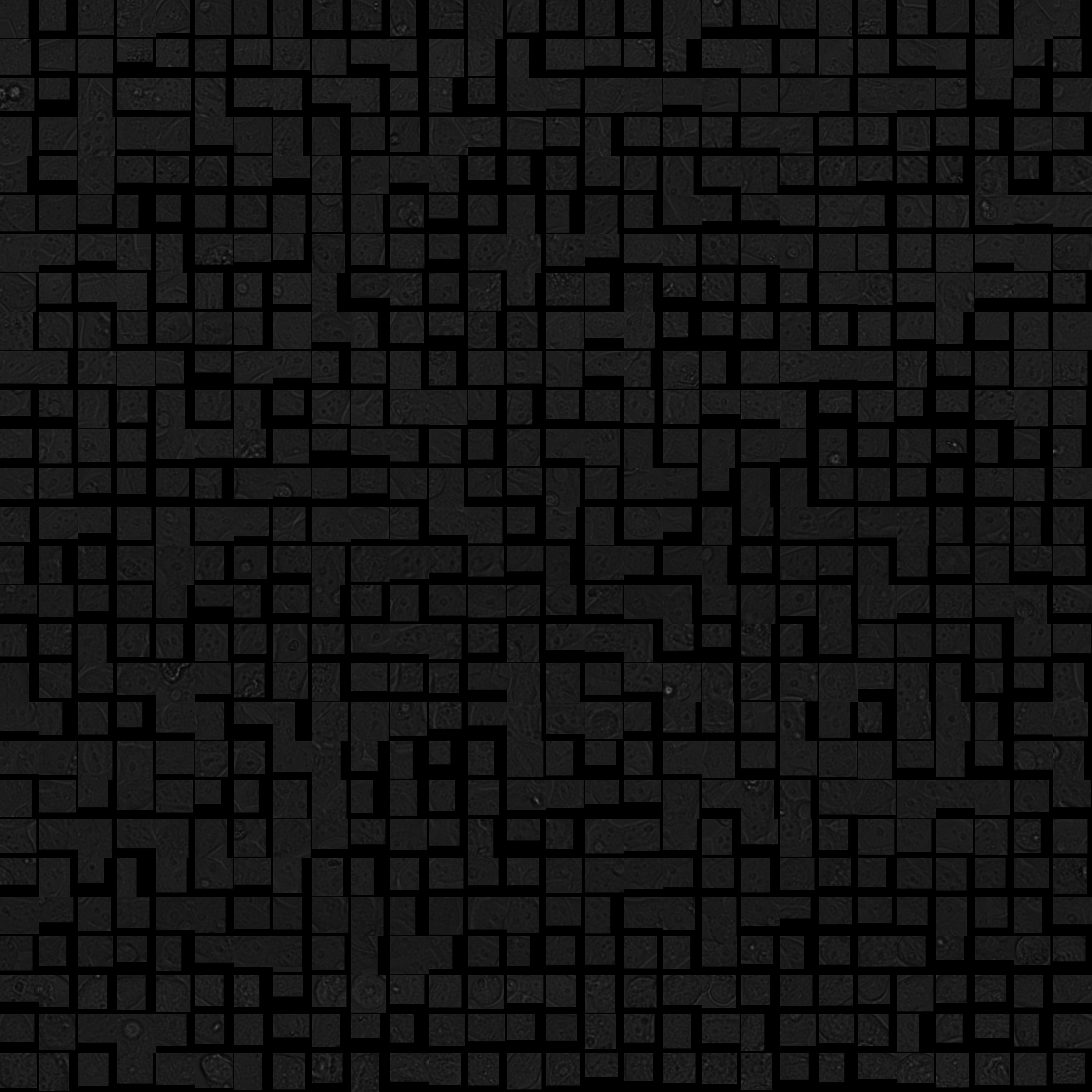

Supplement: Supplementary file 10 — Supplementary Data 8 [file 41540_2021_190_MOESM10_ESM.bz2 › D2_Additional_file_4_SupDataS2/genlarge-08.tif]

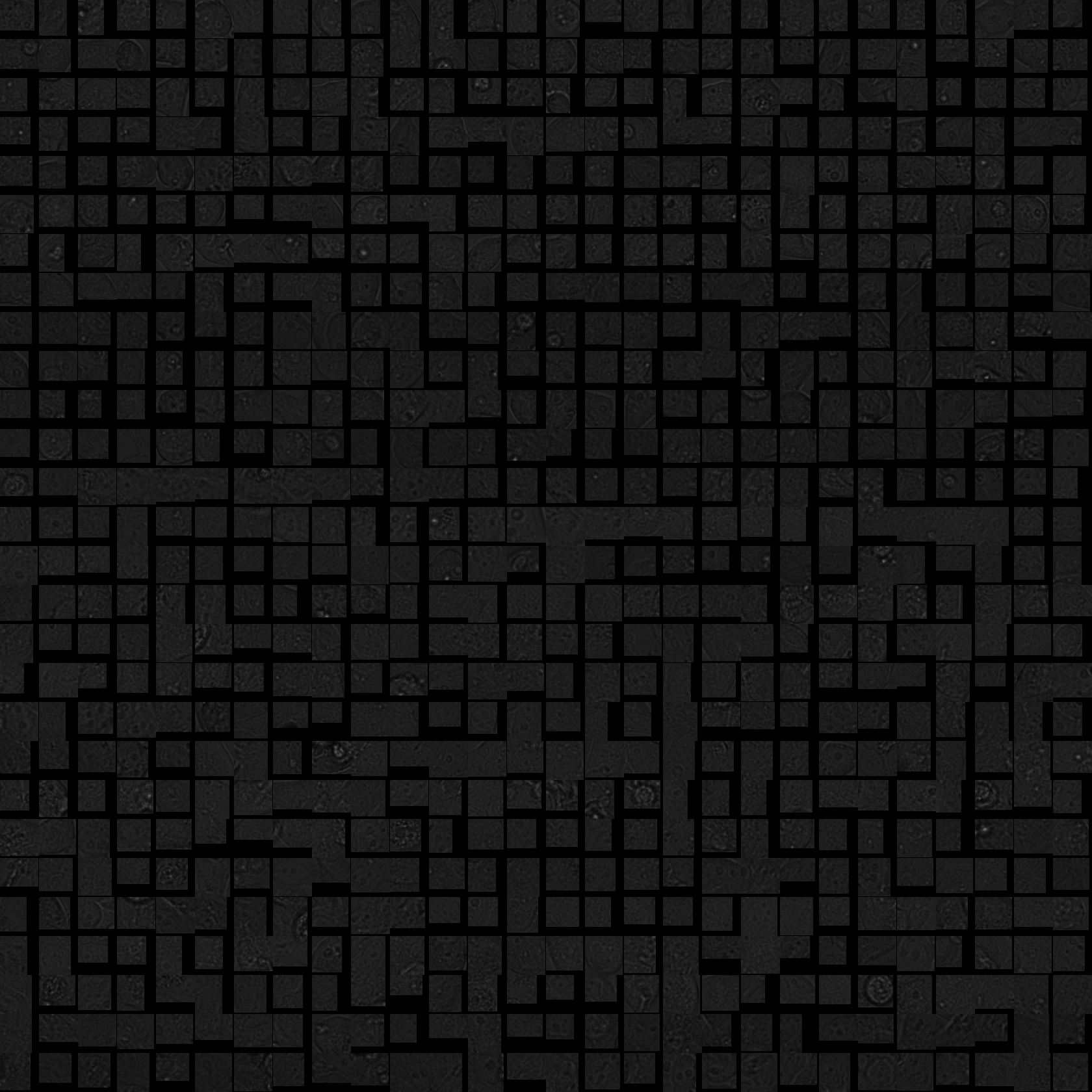

Supplement: Supplementary file 10 — Supplementary Data 8 [file 41540_2021_190_MOESM10_ESM.bz2 › D2_Additional_file_4_SupDataS2/genlarge-09.tif]

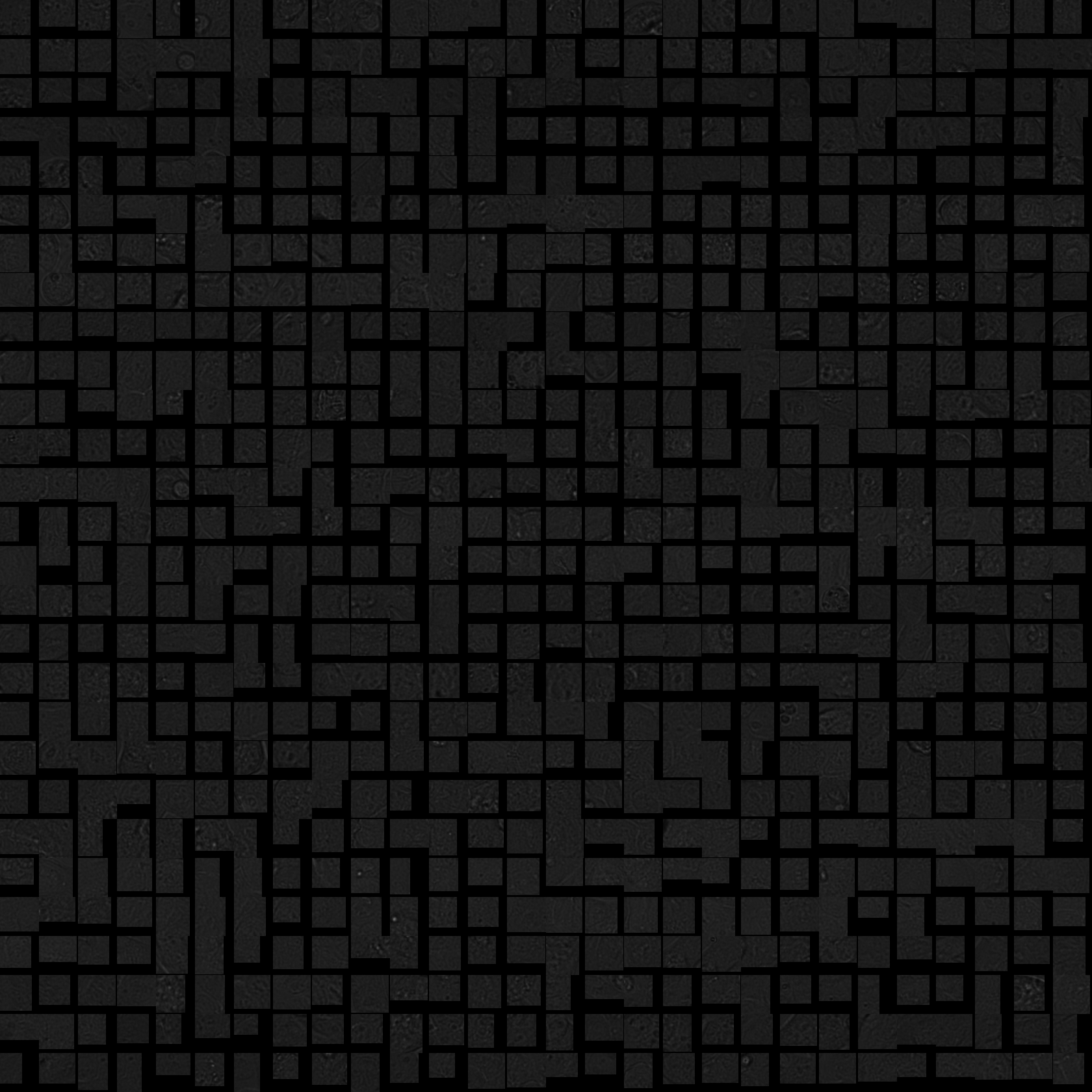

Supplement: Supplementary file 10 — Supplementary Data 8 [file 41540_2021_190_MOESM10_ESM.bz2 › D2_Additional_file_4_SupDataS2/genlarge-10.tif]

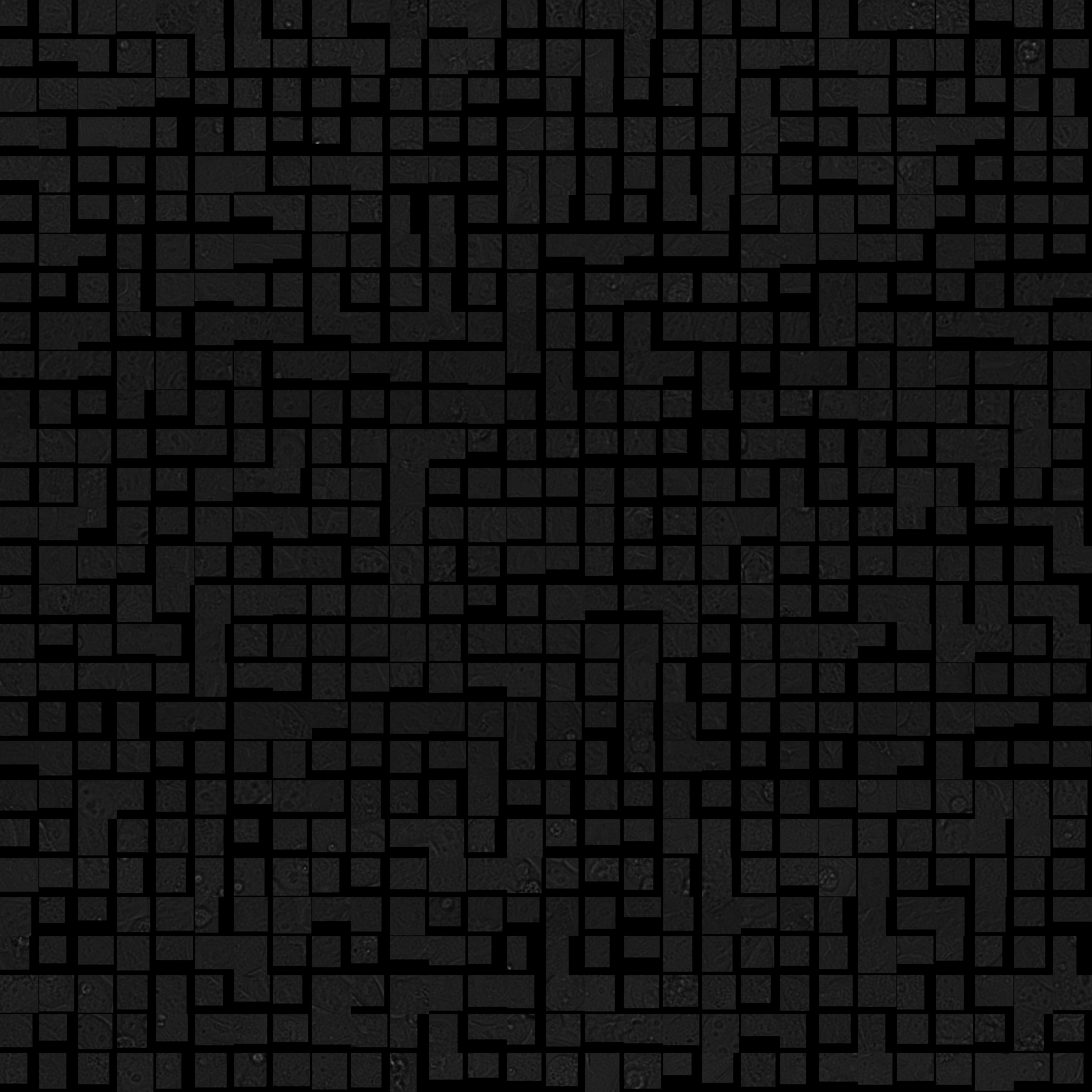

Supplement: Supplementary file 10 — Supplementary Data 8 [file 41540_2021_190_MOESM10_ESM.bz2 › D2_Additional_file_4_SupDataS2/genlarge-11.tif]

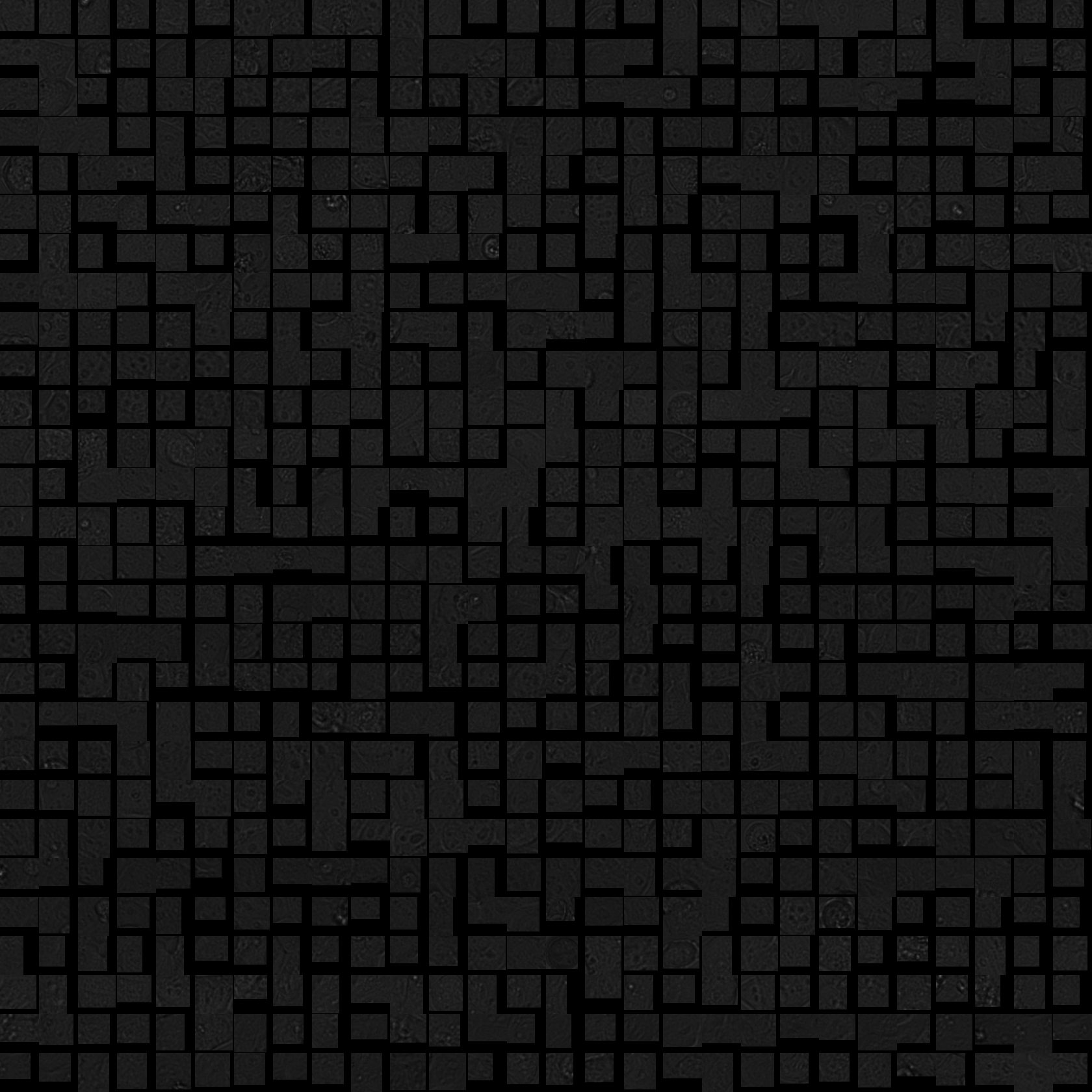

Supplement: Supplementary file 10 — Supplementary Data 8 [file 41540_2021_190_MOESM10_ESM.bz2 › D2_Additional_file_4_SupDataS2/genlarge-12.tif]

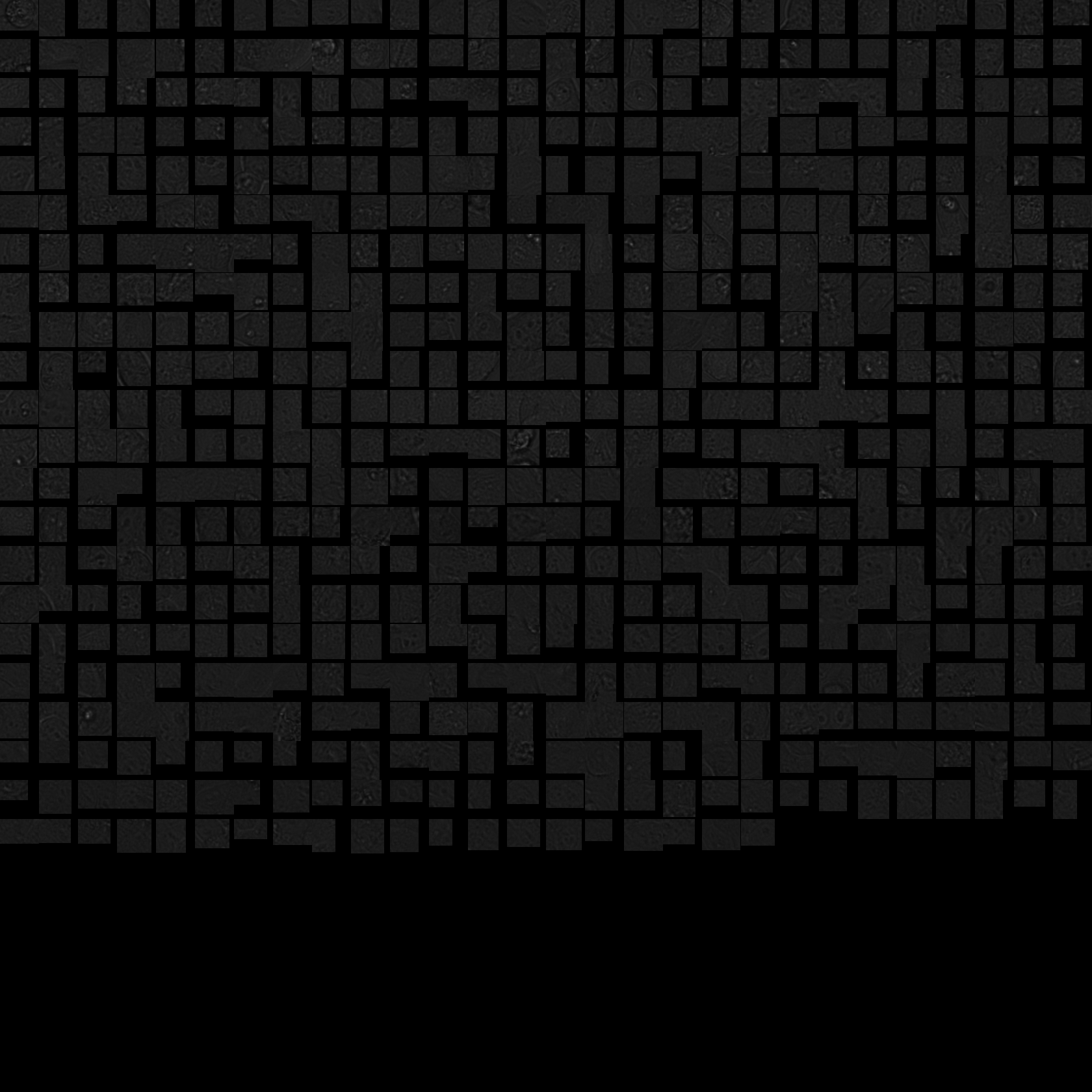

Supplement: Supplementary file 10 — Supplementary Data 8 [file 41540_2021_190_MOESM10_ESM.bz2 › D2_Additional_file_4_SupDataS2/genlarge-13.tif]
